# Effect of Hydrolyzable Tannins on Glucose-Transporter Expression and Their Bioavailability in Pig Small-Intestinal 3D Cell Model

**DOI:** 10.3390/molecules26020345

**Published:** 2021-01-11

**Authors:** Maksimiljan Brus, Robert Frangež, Mario Gorenjak, Petra Kotnik, Željko Knez, Dejan Škorjanc

**Affiliations:** 1 Faculty of Agriculture and Life Sciences, University of Maribor, Pivola 10, 2311 Hoče, Slovenia; maksimiljan.brus@um.si; 2 Veterinary Faculty, Institute of Preclinical Sciences, University of Ljubljana, Gerbičeva 60, 1000 Ljubljana, Slovenia; robert.frangez@vf.uni-lj.si; 3 Center for Human Molecular Genetics and Pharmacogenomics, Faculty of Medicine, University of Maribor, Taborska 8, 2000 Maribor, Slovenia; mario.gorenjak@um.si; 4 Department of Chemistry, Faculty of Medicine, University of Maribor, Taborska 8, 2000 Maribor, Slovenia; petra.kotnik@um.si (P.K.); zeljko.knez@um.si (Ž.K.); 5 Laboratory for Separation Processes and Product Design, Faculty of Chemistry and Chemical Engineering, University of Maribor, Smetanova 17, 2000 Maribor, Slovenia

**Keywords:** hydrolyzable tannin, glucose, glucose transporters, porcine epithelial cell line, tannin bioavailability, glucose transport

## Abstract

Intestinal transepithelial transport of glucose is mediated by glucose transporters, and affects postprandial blood-glucose levels. This study investigates the effect of wood extracts rich in hydrolyzable tannins (HTs) that originated from sweet chestnut (*Castanea sativa* Mill.) and oak (*Quercus petraea*) on the expression of glucose transporter genes and the uptake of glucose and HT constituents in a 3D porcine-small-intestine epithelial-cell model. The viability of epithelial cells CLAB and PSI exposed to different HTs was determined using alamarBlue^®^. qPCR was used to analyze the gene expression of SGLT1, GLUT2, GLUT4, and POLR2A. Glucose uptake was confirmed by assay, and LC–MS/ MS was used for the analysis of HT bioavailability. HTs at 37 µg/mL were found to adversely affect cell viability and downregulate POLR2A expression. HT from wood extract Tanex at concentrations of 4 µg/mL upregulated the expression of GLUT2, as well as glucose uptake at 1 µg/mL. The time-dependent passage of gallic acid through enterocytes was influenced by all wood extracts compared to gallic acid itself as a control. These results suggest that HTs could modulate glucose uptake and gallic acid passage in the 3D cell model.

## 1. Introduction

Finding a suitable natural bioactive substance that could effectively replace banned nutritive antibiotics is crucial to animal breeders, feed producers, and consumers [1]. The bioaccessibility of active nutrient components in the gastrointestinal tract, their bioavailability to the organism, and their activation of organic systems could positively affect animal performance [2].

Tannins are secondary water-soluble phenolic metabolites found in different families of higher plants [3]. Chemically, tannins can be classified as hydrolyzable tannins (HTs), which can be hydrolyzed to gallic acid and glucose, and condensed tannins (CTs), which are composed of flavonoids [4,5]. HTs are polyesters with organic acid and sugar moieties. The sugar component is usually glucose, and if the organic acid is gallic or ellagic acid, compounds are called gallotannins or ellagitannins. These compounds can be hydrolyzed using dilute acids, which cause hydrolytic cleavages of the respective sugar and acid moieties [6].

Tannins maintain bioactive potential, reflected in their antimicrobial [7] and antiviral activity [8]. Some HTs were shown to have an anticancerogenic [9] and positive proliferative effect on untransformed epithelial cell lines in pigs and humans [10]. This efficacy is important for the restoration, development, and maintenance of the small intestine’s absorptive surface. By contrast, little is known about tannins regarding their mechanisms of action and movement across cellular monolayers. The intestinal uptake of tannin metabolic products, such as urolithin A and B, and subsequent action has been established [7]. The molecules of gallic acid are small basic building units of gallotannins and ellagitannins. Currently, it is not known how many of these molecules pass through the enterocyte monolayer.

During digestion, tannin molecules break down into different structural units, such as monomers, dimers, trimers, and polymers, and reach the intestinal mucosa, which is covered by mucin. Mucin acts as a separator of tannin molecules, binding the larger molecules while smaller ones pass by. The flavonol quercetin passes the human intestinal epithelium and does not require active transport [11]. Another mechanism of action was found for proanthocyanidins. By increasing their complexity (dimers, trimers, and polymers), their passage through the cell monolayer was reduced, and their cell-membrane adsorption was increased under in vitro conditions [12]. It is assumed that the passage of polyphenols through the lipid bilayer is inversely proportional to the number of hydroxyl groups, and corresponds to the hydrophobicity of the molecule [13]. HT activity and passage in the digestive tract depend on their chemical characteristics and the complexity of the molecules. In saliva and mucin, tannins associate with protein-soluble and -insoluble complexes. Several factors play an essential role in the formation of tannin/protein complexes, such as the concentration and distribution of proteins, the concentration and diversity of tannins, tannin molecular weight, and the protein:tannin ratio [14]. The potential binding of tannins to molecules is associated with so-called nonspecific forces, such as hydrogen bonding and hydrophobic effects, in addition to formation of covalent bonds. The most likely targets are surface-exposed adhesions, cell-wall polypeptides, and membrane-bound enzymes [15].

A high concentration of plant extract may have a cytotoxic effect on cells. To avoid such situations, a critical concentration of plant extract can be determined in vitro on the basis of POLR2A expression. The housekeeping gene RNA polymerase II POLR2A encodes the large subunit of POLR2A that functions in transcribing protein-coding genes in eukaryotes and plays an essential role in cell survival [16,17]. 

Glucose uptake and transportation across intestinal epithelial cells are also of pivotal importance for postprandial glucose levels. In the small intestine, sodium-glucose cotransporter 1 (SGLT1) mediates almost all sodium-dependent glucose uptake across the apical enterocyte membrane and basolaterally, and glucose leaves the enterocyte through facilitated glucose transporter 2 (GLUT2) [18,19].

At the apical membrane of enterocytes, GLUT2 can also be present [20,21]. In the case of absorption, saturation occurs at glucose concentrations of more than 10 mM on the apical side of enterocytes and after prolonged cell exposure [22]. This leads to a redistribution of GLUT2 glucose transporters from the basolateral to the apical surface of epithelial cells [23]. Apical GLUT2 acts in tandem with SGLT1; therefore, it was suggested as a potential therapeutic target for dietary or pharmacological interventions to control intestinal sugar uptake [24,25,26]. Moreover, flavonoids such as quercetin were demonstrated to inhibit SGLT1 and GLUT2 transporters [27,28]. Glucose transporter 4 (solute carrier family 2 member 4; GLUT4) in the cell membrane is essential for the supply of muscle and adipose tissue in mammals. It is involved in the uptake of glucose to muscle and fat tissue cells, and is also present in pig intestine [29]. Condensed tannins and, more specifically, flavonoids, exert in vitro insulin-like effects in insulin-sensitive cells and increase the expression of glucose transporter GLUT4 in cell membranes. Flavonoids can act as an anti-hyperglycemic agent with insulin-mimetic properties [30].

The present study was conducted to investigate the effects of HTs from three commercially available wood extracts (*Castanea sativa* Mill.) with different ellagi- and gallotannin contents on the expression of glucose transporters, and their bioavailability in a porcine enterocyte cell model. It is hypothesized that (i) the presence of tannins in the small-intestinal epithelial cell model affects the expression of glucose transport genes and, thereby, the transfer of essential nutrients such as glucose, and (ii) HTs added to the cell model pass through the apical cell membrane and will be present in the basolateral part of the cell membrane. To test these hypotheses, functional three-dimensional (3D) cell models (CLAB, PSI) of pig small intestine were developed [31,32,33]. Cellular viability was determined on the basis of cytotoxicity analyses using alamarBlue^®^, and genotoxicity with POLR2A. On the basis of the results of both analyses, the range of HT concentrations for further investigation was determined. The effect of different HT concentrations on the gene expression of glucose carriers SGLT1, GLUT2, and GLUT4 in normal porcine enterocytes (CLAB) was estimated, after which the glucose content in the basolateral part of the model was analyzed. For the determination of HT bioavailability, a PSI model of a porcine intestinal cell line was used. The chemical components of all three commercial-feed additives were analyzed using liquid chromatography–mass spectrometry (LC–MS). Gallic acid was selected as the most important basic structural component of tannins for further bioavailability analysis. Lastly, the transport of the gallic acid through the PSI monolayer (apical, intracellular, and basolateral parts) was studied.

## 2. Results

### 2.1. Viability of Cells Exposed to Wood Extracts

**Figure 1:**
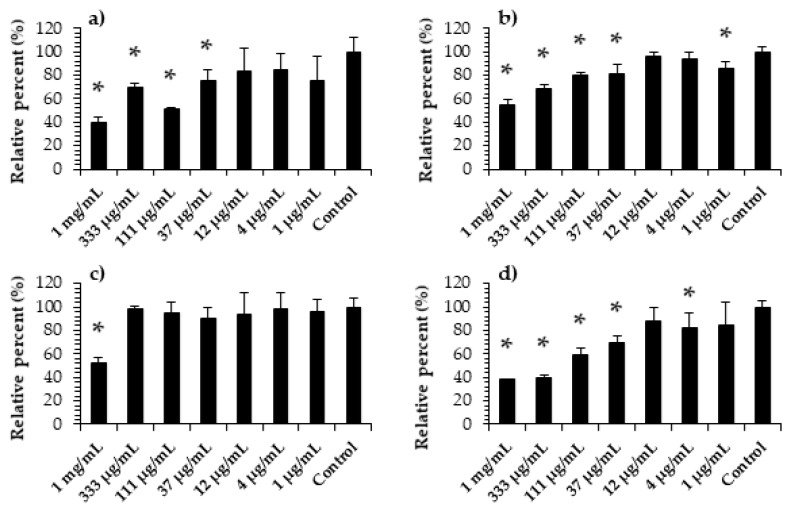
Viability of exposed normal CLAB cells. (**a**) Tanex; (**b**) Farmatan; (**c**) Contan; (**d**) gallic acid. Results presented as mean absorbance/fluorescence values ± standard deviations of independent triplicates expressed as percentage relative to control. * represents statistically significant differences between concentrations and control within the studied HT at *p* ≤ 0.05.

Prior to gene-expression assays, the viability of CLAB cells exposed to wood-extract suspensions at different concentrations, using alamarBlue^®^ as an indicator of cell-viability, was determined. The obtained data show that high concentrations of wood extracts containing HTs adversely affected cellular viability. According to the results of cytotoxicity, these concentrations are greater than 37 µg/mL for Tanex, Farmatan and gallic acid, and Contan, for concentrations greater than 1 mg/mL. The viability of CLAB cells exposed to Tanex was significantly reduced (*p* ≤ 0.05) by 60%, 30%, 49%, and 25%, at concentrations of 1 mg/mL and 333, 111, and 37 µg/mL, respectively, compared to the control group (Figure 1a). Farmatan also significantly reduced (*p* ≤ 0.05) the viability of CLAB cells by 45%, 31%, 20%, 19% and 15% compared to the control group at concentrations of 1 mg/mL, and 333, 111, 37, and 1 µg/mL, respectively (Figure 1b). In contrast to Tanex and Farmatan, the addition of Contan significantly reduced fluorescence at only 1 mg/mL (48%; *p* ≤ 0.05; Figure 1c). The addition of 1 mg/mL and 333, 111, 37, and 4 µg/mL of gallic acid significantly reduced cell viability by 62%, 60%, 41%, 30%, and 17%, respectively (*p* ≤ 0.05; Figure 1d), in comparison to the control group.

### 2.2. Gene Expression in CLAB Cells Exposed to Wood Extracts

Gene-expression analysis was conducted on CLAB cells to investigate the impact of wood extracts on the expression of genes involved in glucose transport. Cell cultures treated with the wood extracts Tanex, Farmatan, and Contan, and gallic acid, were incubated for 6 h at 37 °C and prepared for gene-expression analysis. Unexposed cells were used as controls. Before analyzing the target genes, qPCR melting-curve analysis and end-point PCR were performed to check the primer annealing temperatures and the specificity of the PCR amplicons. In all cases, the PCR amplicons corresponded to a single specific product at an annealing temperature of 60 °C. Various concentrations of HT-rich extract (37, 12, 4, and 1 µg/mL) were selected on the basis of the previous cell-viability assay (Figure 1) and following genotoxicity analysis. The latter was based on the analysis of POLR2A expression, which was performed separately for each extract concentration up to and including the first significant cytotoxic concentration. At 37 μg/mL, Farmatan and Contan downregulated the expression of POLR2A in CLAB cells, with statistically significant differences between the groups (*p* ≤ 0.05) and median Ct values of 3.72 and 1.46. Therefore, the expression data of target genes obtained from cells exposed to 37 μg/mL wood extracts were excluded from further analysis of the CLAB model.

**Figure 2:**
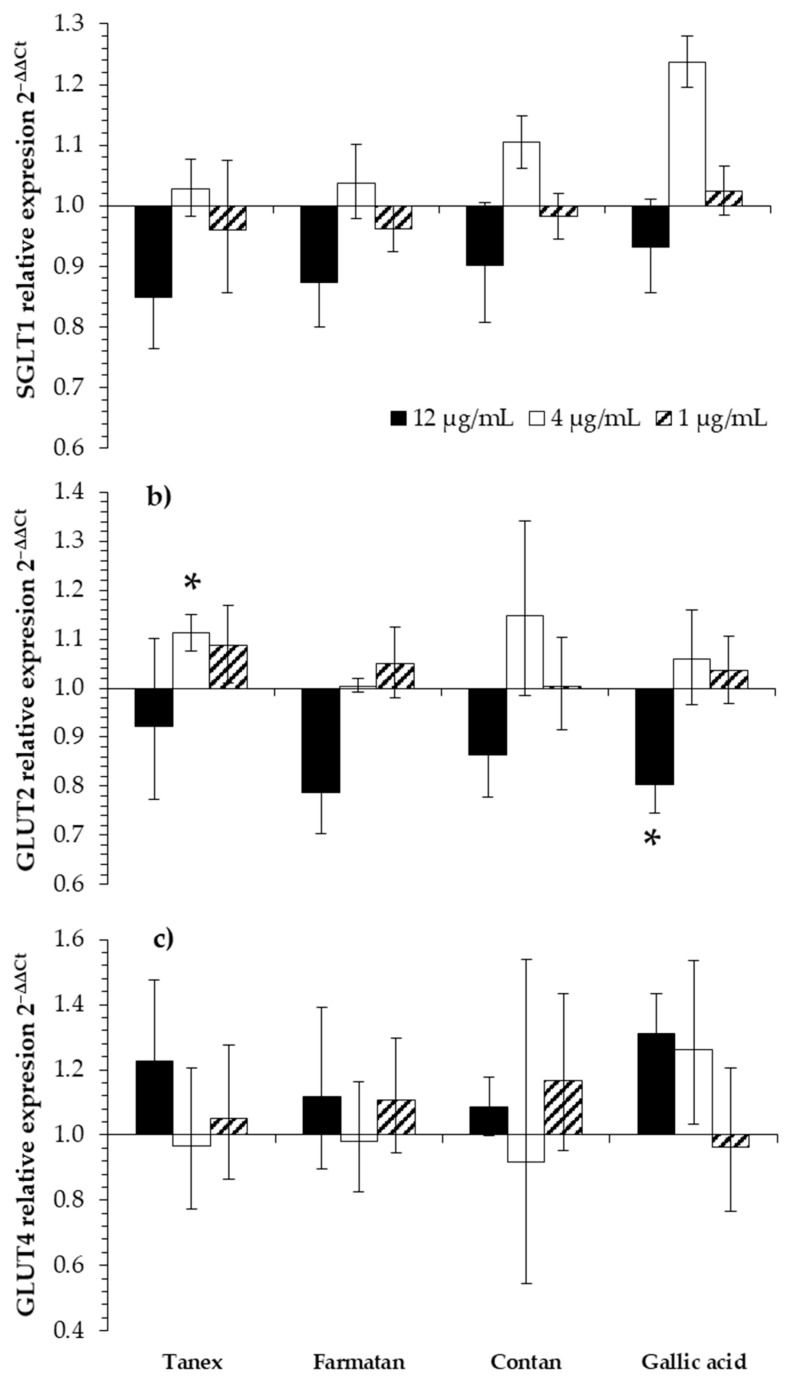
Analysis of SGLT1, GLUT2, and GLUT4 gene expression in CLAB cells exposed to wood extracts for 6 h. (**a**) SGLT1; (**b**) GLUT2; (**c**) GLUT4. Results presented as mean 2^−ΔΔCt^ expression relative to control of independent triplicates ± error of expression calculated from standard deviations. * represents statistically significant differences in gene expression of SGLT1, GLUT2 and GLUT4 between different HT concentrations and control (without added additive) at *p* ≤ 0.05.

Wood extracts notably impacted the expression of target glucose transporter genes in CLAB cells. All wood extracts, including gallic acid at a concentration of 12 µg/mL, showed the ability to downregulate SGLT1 expression. The addition of Tanex, Farmatan, Contan and gallic acid downregulated the expression of SGLT1, but the difference from the control was not significant (Figure 2a). At a concentration of 4 µg/mL, a trend of SGLT1 upregulation was noticed. At lower concentrations (4 µg/mL), Contan and gallic acid upregulated the expression by 10% and 24%, respectively (Figure 2a). At a concentration of 1 µg/mL, the wood extracts had no perceivable impact on SGLT1 expression.

The expression of GLUT2 was also modulated in two distinct ways (Figure 2b). Gallic acid (12 µg/mL) significantly downregulated GLUT2 expression by 20% (*p* ≤ 0.05) compared with the respective control. However, Farmatan and Contan at 12 µg/mL also downregulated the expression of GLUT2 by 21% and 14%, respectively. Gallic acid significantly reduced (*p* ≤ 0.05) GLUT2 expression by 20% at the same concentration, while 4 µg/mL of Tanex significantly upregulated the expression of GLUT2 by 11% (*p* ≤ 0.05). At a concentration of 1 µg/mL, no notable impact of the wood extracts was determined. Concentrations of 4 and 1 μg/mL had a positive effect on the expression of GLUT2.

Additionally, insulin-sensitive GLUT4 was studied, as it is expressed in porcine intestines. The presence of wood extracts and gallic acid did not affect the expression of GLUT4 relative to the control group (Figure 2c). At a concentration of 12 μg/mL, all additives decreased SGLT1 expression. 

The most desirable concentration was demonstrated to be 4 μg/mL on the basis of the increased expression of SGLT1 in cells treated with this concentration, compared with the control cells. At this concentration, the accelerated transfer of glucose into the cell model can be expected. The final concentration of 1 μg/mL, again, lowered SGLT1 expression. At 12 μg/mL, all additives markedly affected the expression of GLUT2. Compared with other treatments, gallic acid significantly downregulated GLUT2 expression (*p* ≤ 0.05). Concentrations of 4 and 1 μg/mL had a positive effect on the expression of GLUT2 transporters. Compared with the other treatments, the addition of 4 μg/mL, Tanex significantly impacted GLUT2 transporter expression. Interestingly, at a concentration of 12 μg/mL, a positive effect was observed on the expression of the GLUT4, which is normally found in muscles and is an insulin-regulated transporter, while the opposite effect was observed for GLUT2. A negative effect on GLUT4 expression was found at a concentration of 4 μg/mL. However, this effect was not established for gallic acid at the 4 μg/mL but, instead, at 1 μg/mL. Additives other than gallic acid upregulated the expression of GLUT4 at a concentration of 1 μg/mL.

### 2.3. Glucose Bioavailability

**Figure 3:**
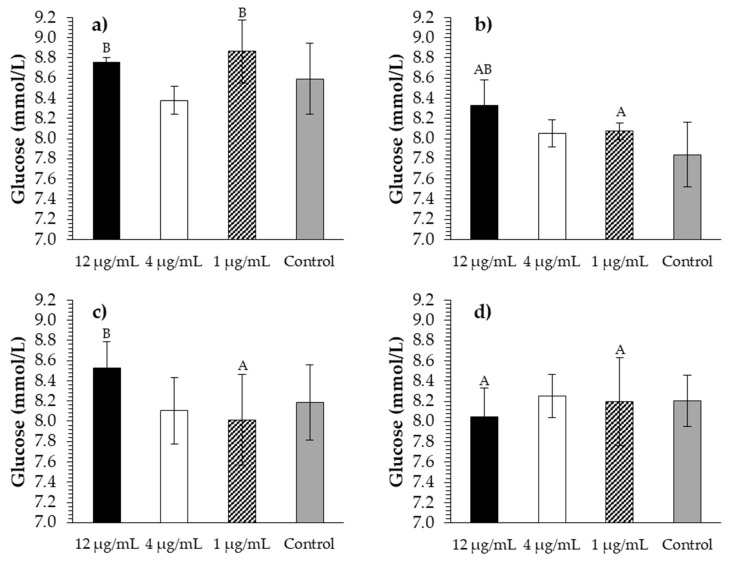
Glucose bioavailability and transport through CLAB cells. (**a**) Tanex; (**b**) Farmatan; (**c**) Contan; (**d**) gallic acid. Results presented as mean glucose concentration of independent triplicates ± standard deviations. Capital letters represent statistically significant differences between HT tannins at *p* ≤ 0.05.

To assess whether the effects of wood extracts on gene expression subsequently affect glucose transport from the apical to the basolateral compartment, a 3D functional CLAB cell model was established. The addition of Tanex, Farmatan, Contan, and gallic acid did not significantly affect glucose transfer in the 3D functional cell model (Figure 3). When using Tanex, the highest glucose concentration was found at 1 μg/mL, and the lowest at 4 μg/mL (Figure 3a). Using Farmatan, the highest glucose concentration in the basolateral part was distinguished when 12 μg/mL was added (Figure 3b). Even at concentrations of 4 and 1 μg/mL, glucose concentration was higher than that of the control group. The maximal glucose concentration in the basolateral part was induced by Contan at 12 μg/mL, and the minimum at 1 μg/mL Contan (Figure 3c). Gallic acid at 12 μg/mL induced the lowest glucose concentration in the basolateral part, and the highest glucose concentrations at 4 and 1 μg/mL, which were similar to control values (Figure 3d). An interesting finding was that glucose transfer was significantly impacted (*p* ≤ 0.05) by the origin of the HTs, and not by the various concentrations used in the experiments. The most effective additive was Tanex. Tanex at a concentration of 1 μg/mL had a statistically significant (*p* ≤ 0.05) facilitating effect on glucose transport compared with the others. At the highest concentration of 12 μg/mL, Tanex and Contan had a statistically significant (*p* ≤ 0.05) facilitating effect on glucose transport compared with gallic acid.

### 2.4. Hydrolyzable-Tannin Bioavailability

**Figure 4:**
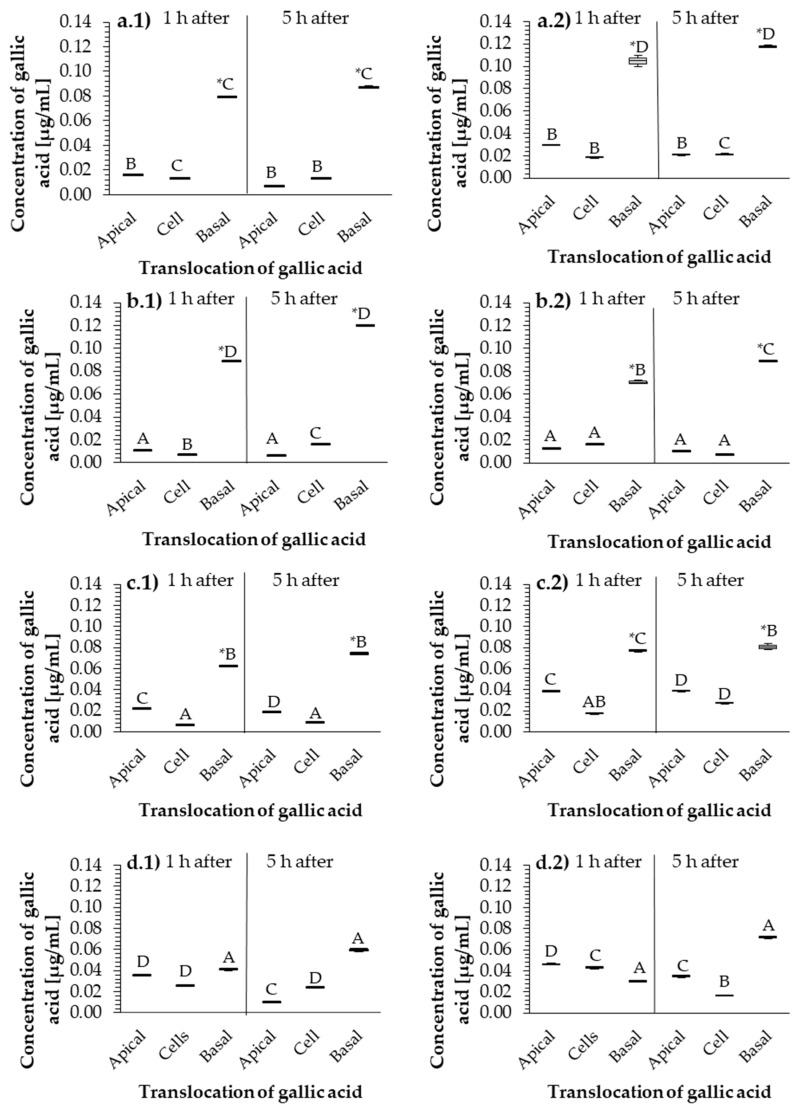
Transport of gallic acid in the presence of (**a**) Tanex, (**b**) Farmatan, (**c**) Contan, and (**d**) gallic acid through PSI cells after 1 and 5 h incubation, and exposed to 12 µg/mL (first column, (**a.1**–**d.1**)) and 37 µg/mL (second column, (**a.2**–**d.2**)). Samples were collected from apical, cell, and basal (basolateral) compartments of the 3D cell model. Capital letters represent statistically significant differences within the same cell compartment between HT tannins at *p* ≤ 0.05. * represents a statistically significant difference between parts of the cell model within a single time interval at *p* ≤ 0.05.

HT bioavailability was determined by measuring the concentration of gallic acid in apical, basolateral, and cell fluid of a 3D cell model. Tests were performed following the addition of Tanex, Farmatan, and Contan extracts and gallic acid at two concentrations (12 and 37 µg/mL) due to the matrix effect on the selectivity of the analytical method. In samples collected after 1 and 5 h incubation, the concentration of gallic acid that passed through the cellular monolayer was determined. Gallic acid concentrations in the apical, basolateral, and cell compartments are presented in Figure 4. Therefore, pure gallic acid was used as the control sample at the same optimal concentrations as those of other additives from the previous experiment. 

Statistical analysis of the data presented in Figure 4 was performed in four sequential steps. In the first step, gallic acid concentrations were examined between apical, cell and basolateral model compartments within a given time. The differences found are marked with an asterisk in Figure 4. A significant (*p* ≤ 0.05) maximum gallic acid concentration was found in the basolateral compartment of the cellular model in Tanex (Figure 4(a.1,a.2)), Farmatan (b.1,b.2), and Contan (c.1,c.2), but no significant (*p* > 0.05) differences were found when pure gallic acid was used as an additive in the 3D cellular model (Figure 4(d.1,d.2)).

In the second step, the concentrations of gallic acid in the same cell compartment were compared separately, after 1 and 5 h, within the concentration of the added HT for each supplemented HT. No significant (*p* > 0.05) differences in gallic acid concentrations were found between the same compartments of the 3D cellular model and the different exposure times at both 12 and 37 μg/mL concentrations. 

In the third step, gallic acid concentrations were compared in the same cell compartment at the same time but between different concentrations of the same HT supplement. Statistical analysis of gallic acid concentrations showed no significant (*p* > 0.05) differences for each cell compartment of the 3D cell model. 

In the fourth step, gallic acid concentrations were compared within the same cell compartment, time, and HT concentration, but between different HTs. Statistically significant differences were represented by capital letters A, B, C and D. Gallic acid passes most rapidly from the apical compartment in Farmatan (Figure 4(b.1)) to the cellular compartment in Contan (Figure 4(c.1)) during the first hour at 12 μg/mL. At this concentration, pure gallic acid is released most slowly from both the apical and cellular compartments of the 3D model (Figure 4(d.1)). The highest concentration of gallic acid (*p* ≤ 0.05) is measured in the basolateral compartment in Farmatan (Figure 4(b.1)). A very similar pattern of gallic acid passage at the same concentration is also found when sampled at five hours. Significantly (*p* ≤ 0.05), however, the highest concentration of gallic acid was found in the basolateral compartment in Tanex (Figure 4(a.2)) and the lowest when pure gallic acid was added (Figure 4(d.2)).

## 3. Discussion

### 3.1. Gene Expression and Glucose Transport 

The results demonstrate that wood extracts (Tanex, Farmatan, Contan) rich in HTs could modulate the expression of POLR2A and glucose-transporter genes in a 3D porcine intestinal cell model. HTs at certain concentrations adversely affect cell viability and modulate the expression of POLR2A. Therefore, concentrations higher than 37 µg/mL that affected the POLR2A genes were not suitable for in vitro experiments in the CLAB or PSI enterocyte cell model.

Glucose from the intestinal lumen is absorbed primarily in the apical membrane by SGLT1, and by GLUT2 at the basolateral part of enterocytes, into the interstitial fluid [18,19]. Absorption saturation occurs at glucose concentrations of more than 10 mM following prolonged cell exposure on the apical side of the enterocytes [22]. This leads to a redistribution of glucose transporter GLUT2 from the basolateral to the apical part of epithelial cells [23]. 

A number of factors can influence glucose transport. In the Caco-2 cell line, glucose transport is inhibited in the presence of polyphenols and phenolic acids from fruit extracts [34]. In contrast to the results of this study, a report of work in rat enterocytes showed greater inhibition of GLUT2 expression than of SGLT1, and plant extracts rich in polyphenols were able to reduce SGLT1 gene expression [35]. Quercetin, which belongs to a specific group of condensed tannins (CTs) [4,5], also reduces glucose uptake through competitive inhibition of SGLT1 in porcine jejunum and noncompetitive inhibition of GLUT2 in Caco-2E cells [27,28].

The GLUT4 transporter is a vital glucose carrier in muscle. The expression of GLUT4 is induced mainly under the influence of insulin, but a particular response to HTs has been shown (Figure 2c). This insulin mimicking effect has previously been described [36]. However, HT concentrations of 1 and 4 μg/mL could have a notable physiological effect on glucose transport through the monolayer of porcine enterocytes. These findings are well in line with the idea and purpose of using phytogenic additives in animal nutrition [2]. Obtained results suggest that the composition of used additives is important, with various quantities of individual molecules in the form of ellagi- and gallotannins, which could affect glucose transfer. Other studies also reported that insulin-like absorption stimulation of ellagi- and gallotannins enhances glucose uptake by activating the GLUT4 carrier [37,38]. The adipocyte cell model 3T3-L1 cell showed that α-PGG, a common form of HT, stimulates the transfer of GLUT4 from the cytosol to the cell membrane by inducing tyrosine phosphorylation of the insulin receptor within minutes, and increases glucose transfer into the cells by 20–30% [38]. A similar mechanism has been described for tannic acid. In identical cells, tannic acid with 40 mg/L increases glucose uptake [39]. The concentration of 10 µM gallic acid proved to be effective, which led to the displacement of GLUT4 to the surface of the plasma membrane in 3T3-L1 cells.

Nevertheless, increased glucose transport to the muscle is triggered by an insulin-stimulated transfer pathway [40]. Similar findings were obtained in the porcine intestinal model (CLAB) to those in the described adipocyte model [40]. The HT-differentiated supplements of 1 and 4 µg/mL affected glucose transport, but epithelial cells (CLAB model) secreted mucin on its apical surface. Mucin represents highly glycosylated proteins (glycoconjugates) produced by epithelial cells that form a kind of chemical barrier. This could be the reason for the possibly lower cell response and lower glucose transport with respect to gene expression [2,12].

### 3.2. Tannin Bioavailability 

The importance of phytogenic feed additives, such as HTs, is to increase their bioavailability to the animal, which is reflected in higher animal productivity and more efficient production [2]. After ingestion, HTs are hydrolyzed into smaller units [41]. The products of hydrolysis are free gallic and ellagic acid, and gallo- and ellagitannins, which are subjected to further microbial metabolism and absorption [12].

The bioavailability of HTs was determined by measuring the concentration of gallic acid in the apical and basolateral compartments, and in the cell fluid of the cell model. Analyses of HT bioavailability were performed in the PSI cell model with transepithelial electric resistance (TEER) exceeding 600 Ω/cm^2^. High TEER prevented the passive passage of smaller and larger molecules of additives paracellularly through the cell monolayer. It has been suggested that some concentration of gallic acid could bind to a membrane, protein, or DNA, as has been reported for ellagic acid [9]. Gallic acid could also be broken down during transport [42]. The presence of mucin on the epithelial cell surface may bind tannins, which may have a selective effect on the passage of HTs [42,43]. For proanthocyanidins, a similar TEER was developed in the Caco-2 line. With the increasing complexity of polyphenols, it was shown that polyphenols reduce their transition from the apical to the basolateral direction, and increase adsorption at the cell surface, emphasizing that the decisive factor is Ca^2+^, which is known to affect TEER [12]. 

The results of this study demonstrate the rapid passage of gallic acid, with a molecular mass of 170.12 g/mol, through the cell model alone, which occurred within 1 h. This passage time is similar to that in a study in which transition took 2–3 h [44]. Similarly, the rapid passage of small, rather than polymerized, polyphenols through the Caco-2 cell model was detected, but the crucial role of TEER was again emphasized [44,45]. Molecules with a molecular weight of ~1740 g/mol bind to the cell membrane due to their affinity for proteins. Results of the present study are in line with the results of studies mentioned above and are consistent with the mechanism of tannin interactivity along with the adaptation of transepithelial proteins on the cell membrane and the effect of mucin in separating tannins [13,14,15]. Antimutagenic and antitoxic effects of gallic acid have been found at levels above 0.002 mg/mL [45]. This fact is important for future research on tannins as feed supplements used in animal nutrition.

In summary, our results suggest that concentrations of HTs exceeding 37 µg/mL influence the expression of POLR2A genes, and are not suitable for in vitro experiments in a CLAB or PSI porcine enterocyte cell model. Our results indicate that the optimal concentrations of HT for in vitro experiments with porcine enterocyte cell models are 1, 4, and 12 μg/mL, which significantly influence the expression of glucose carriers SGLT1, GLUT2, and GLUT4 in the CLAB model. The bioavailability of HT was analyzed using gallic acid as an important small unit of HT. Gallic acid passed the PSI enterocyte cell monolayer in the first hour and reached the basolateral part. It was concluded that the porcine enterocyte cell model is a suitable tool to study the different effects of feed additives in animal nutrition. Further in vitro investigations of the mechanism by which gallotannins and ellagitannins act may help us to understand their role in vivo.

## 4. Materials and Methods

### 4.1. Cell Cultures

Two noncancerogenic porcine-derived enterocyte cell lines (CLAB and PSI) [31] were grown in 25 cm^2^ cell-culture flasks (Corning Inc, Tewksbury, MA, USA) using Advanced Dulbecco’s Modified Eagle’s Medium (DMEM; Life Technologies, Carlsbad, CA, USA) supplemented with 100 IU/mL penicillin (Sigma, Steinheim, Germany), 0.1 mg/mL streptomycin (Sigma, Steinheim, Germany), 2 mM L-glutamine (Life Technologies, Bleiswijk, The Netherlands), and 5% fetal bovine serum (FBS; Life Technologies Ltd., Paisley, UK). Cells were incubated under controlled humidified conditions with 5% CO_2_ at 37 °C. The medium was changed as necessary until confluent cell monolayers were obtained.

### 4.2. Hydrolyzable Tannins

Commercially available wood extracts rich in HT were obtained from Tanin d.d. Sevnica (Sevnica, Slovenia). Tanex and Farmatan originate from sweet chestnut wood (*Castanea sativa* Mill.), and Contan from oak (*Quercus patraea*). Gallic acid (Sigma, Steinheim, Germany) was used as a reference compound. All wood extracts and gallic acid were in powder form before use. The chemical compositions of the wood extracts are summarized in Table 1. Suitable quantities of wood extracts and gallic acid were resuspended in Advanced DMEM supplemented with 2 mM L-glutamine (Life Technologies, Bleiswijk, The Netherlands), vigorously vortexed for 30 min at room temperature to dissolve the powder, and centrifuged at 10,000× *g* for 10 min. After centrifugation, supernatants were collected and further diluted to the desired concentrations in Advanced DMEM supplemented with 2 mM L-glutamine (Life Technologies, Bleiswijk, The Netherlands).

### 4.3. Viability of Cells Exposed to Wood Extracts

The viability of cells exposed to wood extracts was established using alamarBlue^®^ reagent (Invitrogen Corporation, Carlsbad, CA, USA), which evaluates cell integrity by measuring the reducing power of living cells to convert resazurin to the fluorescent molecule resorufin. CLAB cells were seeded at 25 × 10^3^ cells/well using black flat-bottomed NUNCLON^TM^ (Thermo Scientific, Roskilde, Denmark) 96-well plates. Once seeded, plates were incubated overnight under controlled humidified conditions with 5% CO_2_ at 37 °C to obtain 75% confluence the next day. After appropriate cell monolayers were obtained, cells were washed twice with warm sterile phosphate-buffered saline (PBS, Sigma, Steinheim, Germany) and exposed to 100 µL of wood-extract suspensions. The Tanex, Farmatan, Contan, and gallic acid suspensions were added in triplicate to separate wells at concentrations of 1 mg/mL and 3-fold serial dilutions until 1 µg/mL. Nonexposed cells were used as controls. After the addition of wood-extract suspensions, plates were incubated for 6 h under controlled humidified conditions with 5% CO_2_ at 37 °C. After incubation, the medium was discarded, and 200 µL of fresh Advanced DMEM supplemented with 2 mM L-glutamine (Life Technologies, Bleiswijk, The Netherlands) and 20 µL of alamarBlue^®^ reagent was added. After the addition of the alamarBlue^®^ reagent, plates were placed on a horizontal orbital shaker for 5 min and incubated under controlled humidified conditions with 5% CO_2_ at 37 °C for 1 h. After incubation, the fluorescence of the converted alamarBlue^®^ reagent was measured at an excitation wavelength (EX) of 570 nm and emission wavelength (EM) of 585 nm using a Tecan Infinite M1000 Pro spectrophotometer (Tecan, Maennedorf, Switzerland).

### 4.4. Gene-Expression Assay

CLAB enterocytes were grown as described in Section 4.1. Once confluent, cell monolayers were washed twice with warm 37 °C PBS (Sigma, Steinheim, Germany). Ten milliliters of the Tanex, Farmatan, Contan, and gallic acid suspensions, at concentrations determined with the cell-viability assay, were added in triplicate to separate CLAB cells. In parallel, 10 mL of Advanced DMEM with 2 mM L-glutamine (Life Technologies, Bleiswijk, The Netherlands) was added to three separate wells of CLAB cells, which served as controls. Afterward, cells were incubated under controlled humidified conditions with 5% CO_2_ and 37 °C for 6 h. After incubation, cells were washed with 4 °C PBS, and 1.5 mL of TRIzol reagent (Sigma, Steinheim, Germany) was added to the cell monolayers to extract RNA according to the manufacturer’s instructions.

### 4.5. RNA Concentration, Purity, and Transcription into cDNA

Extracted RNA was analyzed using a NanoDrop 2000 spectrophotometer (Thermo Scientific). Concentration and purity were measured with optical-density readings at 260 and 260/280 nm, respectively. Total (2 µg) RNA extracted from each sample was transcribed into cDNA using a high-capacity cDNA reverse-transcription kit (Applied Biosystems, Carlsbad, California) according to the manufacturer’s instructions. Two microliters of 20-fold-diluted cDNA (5 ng/mL) was used for quantitative real-time PCR (qPCR) gene-expression analysis.

### 4.6. Primer Design and RNA Sequence Retrieval

The RNA sequences used for primer design were retrieved from PubMed Nucleotide (www.ncbi.nlm.nih.gov/nuccore/). Primers were hand-picked and analyzed with the IDT OligoAnalyzer (http://eu.idtdna.com/calc/analyser). All primer pairs were ordered from Sigma. Sequence accession numbers and primer sequences are listed in Table 2.

### 4.7. Quantitative Real-Time PCR

Prior to qPCR analysis, the annealing temperatures of the primers and the specificity of the PCR amplicons were checked using gradient end-point PCR (Biometra, Goettingen, Germany) and agarose gel electrophoresis. qPCR was carried out using a LightCycler 480 (Roche, Basel, Germany) and 2× Maxima SYBR Green qPCR Master Mix (Thermo Scientific, Bleiswijk, The Netherlands) according to the manufacturer’s instructions. Primer concentration was 0.3 µM of each primer, and annealing temperature was set to 60 °C for all primer pairs. After each qPCR assay, melting curves were obtained to confirm the specificity of amplification. Raw qPCR data were normalized using the POLR2A reference gene, and relative gene expression was calculated with the 2^−ΔΔCt^ method as previously described [46].

### 4.8. Glucose Bioavailability

To assess glucose transport through enterocytes, 3D functional cell models were established. CLAB cells were seeded at 5 × 10^5^ cells/insert on the apical compartment with a 0.4 µm porous membrane in 12-well plates (Corning Inc., Tewksbury, MA, USA) using Advanced DMEM supplemented with 100 IU/mL penicillin (Sigma, Steinheim, Germany), 0.1 mg/mL streptomycin (Sigma, Steinheim, Germany), 2 mM L-glutamine (Life Technologies, Bleiswijk, The Netherlands), and 5% FBS (Life Technologies Ltd., Paisley, UK). A total of 1.5 mL of the same medium was added to the basolateral compartments. Plates were incubated under controlled humidified conditions at 5% CO_2_ and 37 °C until the cells had developed a TEER of ~500 Ω. The medium was changed regularly every 3 days or less, depending on cell growth, in order to avoid color change indicating acidic pH. After the development of an appropriate TEER, the apical and basolateral compartments were washed twice with warm sterile PBS. The dissolved Tanex, Farmatan, Contan, and gallic acid powders were further diluted to the desired concentrations in low-glucose DMEM without phenol red (Sigma, Steinheim, Germany), supplemented with 2 mM L-glutamine and 10 mM D-glucose (Sigma, Steinheim, Germany), and 0.5 mL of the suspensions was added in triplicate to separate insert wells. A total of 1.5 mL of low-glucose DMEM without phenol red (Sigma, Steinheim, Germany) and supplemented with only 2 mM L-glutamine, was added to the basolateral compartments. For 12-well plate, 0.5 mL of low-glucose DMEM without phenol red, supplemented with 2 mM L-glutamine and 10 mM D-glucose (Sigma, Steinheim, Germany), was added to each well. Experiments were performed in triplicate in separate insert wells, which served as controls for each set of experiments/plate. Plates were then incubated under controlled humidified conditions at 5% CO_2_ and 37 °C for 6 h. After incubation, the basolateral medium from each well was carefully aspirated, transferred to microcentrifuge tubes, and stirred. Five hundred microliters of stirred basolateral media were placed into 10 kDa ultrafiltration columns (Abcam, Cambridge, UK) and centrifuged at 10,000× *g* for 10 min to deproteinize the samples. After centrifugation, the glucose concentration in filtrated medium was measured using a glucose-assay kit (Abcam, Cambridge, UK) according to the manufacturer’s instructions.

### 4.9. Hydrolyzable-Tannin Bioavailability

To assess HT bioavailability and transport through enterocytes, 3D functional cell models were established. PSI cells were seeded at 15 × 10^4^ cells/insert on the apical compartment with a 0.4 µm porous membrane in 12-well plates (Corning Inc., Tewksbury, Massachusetts) using Advanced DMEM supplemented with 100 IU/mL penicillin (Sigma, Steinheim, Germany), 0.1 mg/mL streptomycin (Sigma, Steinheim, Germany), 2 mM L-glutamine (Life Technologies, Bleiswijk, The Netherlands), and 5% FBS (Life Technologies Ltd., Paisley, UK). A total of 1.5 mL of the same media was added to the basolateral compartments. Plates were incubated under controlled humidified conditions with 5% CO_2_ at 37 °C until cells had developed TEER > 600 Ω. The medium was changed as needed. After the development of an appropriate TEER, the apical and basolateral compartments were washed twice with warm sterile PBS buffer (Sigma, Steinheim, Germany). The supernatants of the dissolved Tanex, Farmatan, Contan, and gallic acid powders were further diluted to the desired concentrations of 1, 4, 12, and 37 µg/mL in Hanks’ Balanced Salt Solution (HBSS). In each 12-well plate, 0.5 mL of each suspension was added to the separated apical compartments of the insert wells in triplicate, and 1.5 mL of HBSS was added to the basolateral compartments. Plates were again incubated under controlled humidified conditions at 5% CO_2_ and 37 °C for 1, 2.5, and 5 h. After incubation, the apical and basolateral media from the initial treatment were each aspirated and transferred to microcentrifuge tubes. Immediately after sampling, samples were frozen at −80 °C until further analysis. After carefully aspirating the media from the apical and basolateral compartments, compartments were washed twice with warm sterile PBS. A suspension of 0.5 mL of PBS and 0.5% Triton X-100 (Sigma-Aldrich, Saint Louis, MO, USA) was then added to the same separated apical compartments of the insert wells in triplicate. Plates were again incubated under controlled humidified conditions with 5% CO_2_ at 37 °C for 0.5 h. After incubation, the medium from each well was carefully aspirated and transferred to microcentrifuge tubes. Immediately after collection, samples were deep-frozen at −80 °C until further analysis.

### 4.10. LC–MS/MS Analysis of Hydrolyzable Tannins

An Agilent 1200 HPLC system in tandem with an Agilent 6460 triple quadrupole MS system (Agilent Technologies, Inc., Santa Clara, CA, USA) was used for the separation and determination of tannins in the samples. The Agilent 1200 HPLC system was equipped with a quaternary pump (maximal pressure, 400 bar), an autosampler, and a column thermostat (maximal temperature, 80 °C). An Agilent JetStream technology ionization source was employed for sample analysis. The apparatus was controlled by Agilent MassHunter Workstation software version 6.0, with which the qualification and quantification of gallic acid were performed.

For the quantification of the selected tannins, the mass spectrometer was operated at full and product-ion scan modes with a dwell time of 200 ms, the fragmenter was set to 45 V, and the collision energies were 12 and 36 V. Optimization for gallic acid was carried out using Agilent MassHunter Optimizer Automated Method Development software (version B.02.01) after injecting an analytical standard. Data processing was done using Agilent MassHunter Workstation software, version 6.0, which was used to identify and quantify HT residues.

Unfrozen samples were directly injected (volume of 5 µL) into an analytical Poroshell 120-EC C18 column (100 mm × 2.1 mm) with 2.7 μm particles. The column was maintained at 40 °C; sample separation was achieved by using a 0.3 mL/min flow rate using a gradient from 2% to 80% mobile phase B. Mobile phase A consisted of water with 0.1% formic acid, and mobile phase B consisted of methanol with 0.1% formic acid. The gradient method was composed of multiple linear gradients as follows: 0 min, 2% B; 5 min, 15% B; 30 min, 60% B; 45 min, 80% B; 50 min, 2% B; and 10 min post time. The MS acquisition method applied the following parameters: capillary voltage of 4500 V, nozzle voltage of 1500 V, sheath gas flow of 12 L nitrogen/min at a temperature of 380 °C, drying gas flow of 6 L/min at a temperature of 280 °C, nebulizer gas flow of 35 psi, and quadrupole (Q)1 and Q3 set to a unit resolution of 0.7 full width at half maximum (FWHM) with a dwell time of 200 ms. The identification and quantification of the components were carried out on the basis of transitions (fragmentation and collision energy) in negative ionization: gallic acid 169 → 125 (45 and 12 V).

### 4.11. Statistical Analysis

Obtained data were analyzed using SPSS IBM Statistics version 25.0 (IBM Inc., Armonk, NY, USA) with a nonparametric Mann–Whitney U-test or Kruskal–Wallis test for more than two groups, and a Friedman test for correlated samples. Gene-expression data were analyzed as normalized linear form using 2^−ΔΔCt^ calculations [46]. *p* values ≤ 0.05 were considered to indicate statistically significant differences.

## 5. Conclusions

Based on the results of experiments we conclude the following:

- Tanex at 4 µg/mL significantly increases the expression of GLUT2 in CLAB cells.
- Tanex at 1 µg/mL significantly facilitated glucose transport in the CLAB 3D cell model.
- Glucose uptake in CLAB cells is influenced by the origin of the wood extract.
- Gallic acid passes through the enterocyte in the PSI cell line 3D model, which is most pronounced with Tanex.

## Figures and Tables

**Table 1:** Chemical composition of wood extracts rich with hydrolyzable tannins.

Major Components (%)	Tanex	Farmatan	Contan
Hydrolyzable tannins	63.8	74.3	69.2
Vescalin	0.47	0.9	0.64
Castalin	2.11	1.7	0.94
Roburin A	0.62	0.2	1.86
Gallic acid	2.03	2.4	1.17
Roburin B/C	1.17	2.1	3.57
Grandinin	0.49	0.9	1.21
Roburin D	0.60	1.0	0.26
Vescalagin	0.45	4.7	6.54
Roburin E	2.45	1.5	2.55
Castalagin	2.93	4.1	6.22
Ellagic acid	2.38	0.8	0.42

**Table 2:** Genes, primer sequences, and accession numbers.

Gene	Gene Name	Accession Number	Primer Sequence 5′ → 3′
SGLT1	Solute carrier family 5 member 1	NM_001164021.1	AGTGGGCAGCTCTTCGATTA CCAGCCCAATCATACATCCT Amplicon size: 148 bp
GLUT2	Solute carrier family 2 member 2	NM_001097417.1	ATTCTTTGGTGGGATGCTTG ATGAGATGGTCCCAATTTCG Amplicon size: 118 bp
GLUT4	Solute carrier family 2 member 4	NM_001128433.1	TCATCATCGGCATGAGTTTC CGGGTTTCAGGCACTTTTAG Amplicon size: 121 bp
POLR2A	Polymerase II, polypeptide A	XM_005669224.1	ACCATCAAGCGAGTGCAGTT TCGGTTGTCTCTGGGTATTTG Amplicon size: 95 bp

## Data Availability

The data presented in this study are available on request from the corresponding author. The data is not publicly available because research on the HT operation of this area has been completed, but it is ongoing to provide a complete picture of the HT actions.

## Sample Availability

Samples of the compounds are available from the authors.

## References

[1] Thacker P.A. (2013). Alternatives to antibiotics as growth promoters for use in swine production: A review. J. Anim. Sci. Biotechnol..

[2] Valenzuela-Grijalva N.V., Pinelli-Saavedra A., Muhlia-Almazan A., Dominguez-Diaz D., Gonzalez-Rios H. (2017). Dietary inclusion effects of phytochemicals as growth promoters in animal production. J. Anim. Sci. Technol..

[3] Li M., Kai Y., Qiang H., Dongying J. (2006). Biodegradation of gallotannins and ellagitannins. J. Basic Microbiol..

[4] Khanbabaee K., Van Ree T. (2001). Tannins: Classification and definition. Nat. Prod. Rep..

[5] Mangan J.L. (1988). Nutritional effects of tannins in animal feeds. Nutr. Res. Rev..

[6] Saura-Calixto F., Pérez-Jiménez J., Knasmüller P.D.S., De Marini D.D.M., Johnson P.I., Gerhäuser D.C. (2009). Tannins: Bioavailability and mechanisms of action. Chemoprevention of Cancer and DNA Damage by Dietary Factors.

[7] Smeriglio A., Barreca D., Bellocco E., Trombetta D. (2017). Proanthocyanidins and hydrolysable tannins: Occurrence, dietary intake and pharmacological effects. Br. J. Pharmacol..

[8] Ueda K., Kawabata R., Irie T., Nakai Y., Tohya Y., Sakaguchi T. (2013). Inactivation of pathogenic viruses by plant-derived tannins: Strong effects of extracts from persimmon (*Diospyros kaki*) on a broad range of viruses. PLoS ONE.

[9] Whitley A.C., Stoner G.D., Darby M.V., Walle T. (2003). Intestinal epithelial cell accumulation of the cancer preventive polyphenol ellagic acid—Extensive binding to protein and DNA. Biochem. Pharmacol..

[10] Brus M., Langerholc T., Škorjanc D. Effect of hydrolysable tannins on proliferation of small intestinal porcine and human enterocytes. Proceedings of the 8th International Symposium on the Mediterranean Pig.

[11] Walgren R.A., Walle U.K., Walle T. (1998). Transport of quercetin and its glucosides across human intestinal epithelial Caco-2 cells. Biochem. Pharmacol..

[12] Deprez S., Mila I., Huneau J.F., Tome D., Scalbert A. (2001). Transport of proanthocyanidin dimer, trimer, and polymer across monolayers of human intestinal epithelial Caco-2 cells. Antioxid. Redox Signal..

[13] Tarahovsky Y.S. (2008). Plant polyphenols in cell–cell interaction and communication. Plant Signal Behav..

[14] Soares S., Mateus N., de Freitas V. (2012). Interaction of different classes of salivary proteins with food tannins. Food Res. Int..

[15] Cowan M.M. (1999). Plant products as antimicrobial agents. Clin. Microbiol. Rev..

[16] Cho K.W.Y., Khalili K., Zandomeni R., Weinma R. (1985). The Gene Encoding the large subunit of human RNA polymerase II. J. Biol. Chem..

[17] Ten Asbroek A.L.M.A., Fluiter K., van Groenigen M., Nooij M., Baas F. (2000). Polymorphisms in the large subunit of human RNA polymerase II as target for allele-specific inhibition. Nucleic Acids Res..

[18] Mueckler M. (1994). Facilitative glucose transporters. Eur. J. Biochem..

[19] Wright E.M., Loo D.D.F., Hirayama B.A. (2011). Biology of human sodium glucose transporters. Physiol. Rev..

[20] Ait-Omar A., Monteiro-Sepulveda M., Poitou C., Le Gall M., Cotillard A., Gilet J., Garbin K., Houllier A., Chateau D., Lacombe A. (2011). GLUT2 accumulation in enterocyte apical and intracellular membranes: A study in morbidly obese human subjects and ob/ob and high fat-fed mice. Diabetes.

[21] Kellett G.L., Helliwell P.A. (2000). The diffusive component of intestinal glucose absorption is mediated by the glucose-induced recruitment of GLUT2 to the brush–border membrane. Biochem. J..

[22] Zheng Y., Scow J.S., Duenes J.A., Sarr M.G. (2012). Mechanisms of glucose uptake in intestinal cell lines: Role of GLUT2. Surgery.

[23] Kellett G.L. (2001). The facilitated component of intestinal glucose absorption. J. Physiol..

[24] Kellett G.L., Brot-Laroche E. (2005). Apical GLUT2: A major pathway of intestinal sugar absorption. Diabetes.

[25] Gorboulev V., Schurmann A., Vallon V., Kipp H., Jaschke A., Klessen D., Friedrich A., Scherneck S., Rieg T., Cunard R. (2012). Na(+)-D-glucose cotransporter SGLT1 is pivotal for intestinal glucose absorption and glucose-dependent incretin secretion. Diabetes.

[26] Röder P.V., Geillinger K.E., Zietek T.S., Thorens B., Koepsell H., Daniel H. (2014). The role of SGLT1 and GLUT2 in intestinal glucose transport and sensing. PLoS ONE.

[27] Cermak R., Landgraf S., Wolffram S. (2004). Quercetin glucosides inhibit glucose uptake into brush-border-membrane vesicles of porcine jejunum. Br. J. Nutr..

[28] Kwon O., Eck P., Chen S.L., Corpe C.P., Lee J.H., Kruhlak M., Levine M. (2007). Inhibition of the intestinal glucose transporter GLUT2 by flavonoids. FASEB J..

[29] Aschenbach J.R., Steglich K., Gabel G., Honscha K.U. (2009). Expression of mRNA for glucose transport proteins in jejunum, liver, kidney and skeletal muscle of pigs. J. Physiol. Biochem..

[30] Pinent M., Blay M., Blade M.C., Salvado M.J., Arola L., Ardevol A. (2004). Grape seed-derived procyanidins have an antihyperglycemic effect in streptozotocin-induced diabetic rats and insulinomimetic activity in insulin-sensitive cell lines. Endocrinology.

[31] Cencic A., Langerholc T. (2010). Functional cell models of the gut and their applications in food microbiology—A review. Int. J. Food Microbiol..

[32] Gorenjak M., Skok P., Cencic A. (2012). Novel promising functional cell models to study molecular events in metabolic syndrome. Nutr. Ther. Metab..

[33] Gradisnik L., Filipic B., De Vaureix C., Lefevre F., La Bonnardiere C., Cencic A. (2006). Establishment of a functional cell culture model of the pig small intestine. ALTEX.

[34] Manzano S., Williamson G. (2010). Polyphenols and phenolic acids from strawberry and apple decrease glucose uptake and transport by human intestinal Caco-2 cells. Mol. Nutr. Food Res..

[35] Oliveira D.M., Freitas H.S., Souza M.F.F., Arçari D.P., Ribeiro M.L., Carvalho P.O., Bastos D.H.M. (2008). Yerba mate’(*Ilex paraguariensis*) aqueous extract decreases intestinal SGLT1 Gene expression but does not affect other biochemical parameters in alloxan-diabetic Wistar rats. J. Agric. Food Chem..

[36] Zhang J., Li L., Kim S.H., Hagerman A.E., Lu J. (2009). Anti-cancer, anti-diabetic and other pharmacologic and biological activities of penta-galloyl-glucose. Pharm. Res..

[37] Bai N., He K., Roller M., Zheng B., Chen X., Shao Z., Peng T., Zheng Q. (2008). Active compounds from *Lagerstroemia speciosa*, insulin-like glucose uptake-stimulatory/inhibitory and adipocyte differentiation-inhibitory activities in 3T3-L1 cells. J. Agric. Food Chem..

[38] Cao Y., Himmeldirk K.B., Qian Y., Ren Y., Malki A., Chen X. (2014). Biological and biomedical functions of penta-O-galloyl-D-glucose and its derivatives. J. Nat. Med..

[39] Liu X., Kim J.K., Li Y., Li J., Liu F., Chen X. (2005). Tannic acid stimulates glucose transport and inhibits adipocyte differentiation in 3T3-L1 cells. J. Nutr..

[40] Prasad C.N., Anjana T., Banerji A., Gopalakrishnapillai A. (2010). Gallic acid induces GLUT4 translocation and glucose uptake activity in 3T3-L1 cells. FEBS Lett..

[41] Zanotti I., Dall’Asta M., Mena P., Mele L., Bruni R., Ray S., Del Rio D. (2015). Atheroprotective effects of (poly)phenols: A focus on cell cholesterol metabolism. Food Funct..

[42] Cai K., Hagerman A.E., Minto R.E., Bennick A. (2006). Decreased polyphenol transport across cultured intestinal cells by a salivary proline-rich protein. Biochem. Pharmacol..

[43] Cai K., Bennick A. (2006). Effect of salivary proteins on the transport of tannin and quercetin across intestinal epithelial cells in culture. Biochem. Pharmacol..

[44] Matsui T., Ueda T., Oki T., Sugita K., Terahara N., Matsumoto K. (2001). Alpha-glucosidase inhibitory action of natural acylated anthocyanins. 1. Survey of natural pigments with potent inhibitory activity. J. Agric. Food Chem..

[45] Sevgi K., Tepe B., Sarikurkcu C. (2015). Antioxidant and DNA damage protection potentials of selected phenolic acids. Food Chem. Toxicol..

[46] Livak K.J., Schmittgen T.D. (2001). Analysis of relative gene expression data using real-time quantitative PCR and the 2^−ΔΔCT^ Method. Methods.

